# Anti-Cancer and Electrochemical Properties of Thiogenistein—New Biologically Active Compound

**DOI:** 10.3390/ijms22168783

**Published:** 2021-08-16

**Authors:** Elżbieta U. Stolarczyk, Weronika Strzempek, Marta Łaszcz, Andrzej Leś, Elżbieta Menaszek, Katarzyna Sidoryk, Krzysztof Stolarczyk

**Affiliations:** 1 Research Analytics Team, Analytical Department, Łukasiewicz Research Network—Industrial Chemistry Institute, 8 Rydygiera Street, 01-793 Warsaw, Poland; marta.laszcz@ichp.pl; 2 Faculty of Chemistry, Jagiellonian University, 2 Gronostajowa Street, 30-387 Krakow, Poland; weronika.strzempek@doctoral.uj.edu.pl; 3 Faculty of Pharmacy, Collegium Medicum, Jagiellonian University, 9 Medyczna Street, 30-068 Krakow, Poland; elzbieta.menaszek@uj.edu.pl; 4 Faculty of Chemistry, University of Warsaw, 1 Pasteura Street, 02-093 Warsaw, Poland; ales@chem.uw.edu.pl (A.L.); kstolar@chem.uw.edu.pl (K.S.); 5 Chemistry Group, Department of Pharmacy, Cosmetic Chemistry and Biotechnology, Łukasiewicz Research Network—Industrial Chemistry Institute, 8 Rydygiera Street, 01-793 Warsaw, Poland; katarzyna.sidoryk@ichp.pl

**Keywords:** anticancer drugs, nanoparticles, molecular modeling, oxidation mechanisms, electrochemistry, MALDI, spectroscopic data, cytotoxic study, self-assembled monolayer, gold electrode

## Abstract

Pharmacological and nutraceutical effects of isoflavones, which include genistein (GE), are attributed to their antioxidant activity protecting cells against carcinogenesis. The knowledge of the oxidation mechanisms of an active substance is crucial to determine its pharmacological properties. The aim of the present work was to explain complex oxidation processes that have been simulated during voltammetric experiments for our new thiolated genistein analog (TGE) that formed the self-assembled monolayer (SAM) on the gold electrode. The thiol linker assured a strong interaction of sulfur nucleophiles with the gold surface. The research comprised of the study of TGE oxidative properties, IR-ATR, and MALDI-TOF measurements of SAM before and after electrochemical oxidation. TGE has been shown to be electrochemically active. It undergoes one irreversible oxidation reaction and one quasi-reversible oxidation reaction in PBS buffer at pH 7.4. The oxidation of TGE results in electroactive products composed likely from TGE conjugates (e.g., trimers) as part of polymer. The electroactive centers of TGE and its oxidation mechanism were discussed using IR supported by quantum chemical and molecular mechanics calculations. Preliminary in-vitro studies indicate that TGE exhibits higher cytotoxic activity towards DU145 human prostate cancer cells and is safer for normal prostate epithelial cells (PNT2) than genistein itself.

## 1. Introduction

Genistein (GE) belongs to the group of isoflavones found mainly in soybeans [1,2]. Some studies suggest that frequent consumption of soy-based products in Asian countries reduces the incidence of breast and prostate cancer compared to Western countries. Genistein also has antioxidant, anti-inflammatory, antiangiogenic, pro-apoptotic, and antiproliferative properties, which gives it great potential for use in anti-cancer therapy. It has been shown that genistein may influence the regulation of apoptosis, angiogenesis, metastasis, and various stages of the cell cycle. In addition to acting on transcription factors, genistein also induces stress on the endoplasmic reticulum, which in turn leads to the apoptosis of neoplastic cells. Additionally, it has been shown to induce apoptosis in cancer cells by targeting the PPAR (peroxisome proliferator-activated receptor) signaling cascade. Various molecular mechanisms of genistein in diverse cancer models were described by Tuli et al. [3]. However, its low water-solubility and poor oral bioavailability severely hamper its use as an ingredient for food and the pharmaceutical industries [4]. Therefore, there is an urgent need for the study of a new analog of genistein, which additionally can be promising for an economical and feasible delivery system to enhance the solubility and dissolution rates, and bioavailability of genistein while maintaining its chemical stability [4].

**Figure 1:**
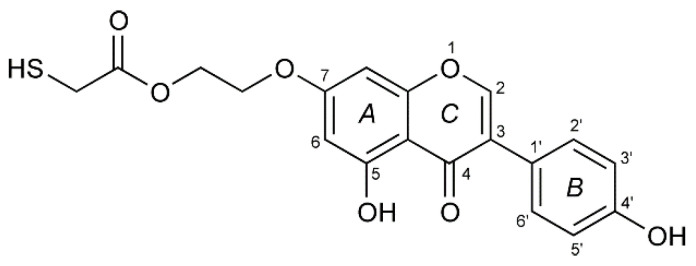
The chemical structure of TGE (the thiolated derivative of genistein). Three rings of the moiety are marked as A, B, and C.

Our previous studies showed that genistein interacts electrostatically with gold nanoparticles [5] which turned out to be interesting carriers for active substances. These studies were continued by Vodnik et al. [6], however the authors did not perform a purity study of conjugates in relation to the free GE and stability of new conjugates as well. It is known that electrostatic interactions are not sufficiently stable at extreme pH and in the presence of salt. For example, Dinkel and co-workers [7] described that citrates are only loosely bound to the gold nanoparticles (6.7 kJ/mol). However, sulfur-containing compounds have been used as excellent ligands for binding to flat gold surfaces as well as gold nanoparticles, because of a very strong interaction of sulfur nucleophiles with gold. Therefore, to increase the strength of the interaction, and improve the stability of AuNPs-GE conjugates, we focused on the investigation of an optimal thiol linker as a new derivative—thiolated genistein (TGE, 7-*O*-[2-(mercaptomethylcarboxy)ethyl-genistein) described by Sidoryk and co-workers [8] (compound No. 26). The structural formula of TGE is presented in Figure 1. TGE is composed of the genistein residue bound at the C7-OH site to the ethyl linker and thioglycolic acid residue. Such a construction assures that the TGE will be bound firmly to the Au surface via the Au-S chemical bond on the gold surface as well as gold nanoparticles.

Because pharmacological and nutraceutical effects of isoflavones are attributed to antioxidant mechanisms also, which protect cells against reducing carcinogenesis, the knowledge of the oxidation mechanisms is crucial to ascertain the influence of the redox behavior, on the pharmacological, nutritional, and chemical properties of our new thiolated analog of genistein. TGE was chemically attached to the gold surface as the self-assembled monolayers (SAMs). The study of SAMs of alkanethiol, alkanedithiol, sulphides, and disulfides on the Au surface allowed us to obtain data about processes occurring at the interface, as well as about interactions between molecules in the monolayer [9].

Aromatic molecules create a highly conjugated system that interacts strongly with the metal surface. Additionally, the steric effect of the aromatic ring may also lead to an increase in molecular disorder within the SAM. Generally, aromatic thiols in SAMs are difficult to study, especially in large systems such as drugs. It is known that SAMs have been used for “underpotential polymerization” of aniline and other compounds on gold electrodes [10]. A multitude of experimental approaches is needed for the characterization of SAMs. Attenuated total reflectance spectroscopy (ATR) is a variant of infrared spectroscopy (IR) and is frequently employed to investigate SAMs. The radiation beam is polarized and reinforced in a perpendicular direction to a surface of a substrate. As a consequence, intensities of absorption bands are strictly related to the orientation of dipoles with respect to the substrate surface. Therefore, a band originating from molecules corresponding to vibrations in the plane perpendicular to the surface will show much greater intensity, than the bands associated with vibration parallel to a surface. This dependence allows for a relatively precise definition of the structure monolayers. Based on the position and intensity of bands, it is possible to obtain information about the degree of monolayer order and the orientation of the molecules that make up the system. In the case of model alkanethiol layers, bands related to stretching vibrations of methyl and methylene groups are used. The ratio of their relative intensities makes it possible to estimate the angle of inclination of the alkyl chains, while their position is closely related to the quasi-crystalline or liquid nature of the monolayer and the presence of gauche defects [11,12,13,14,15,16,17,18,19,20].

Our aim is to study the mechanism of the oxidation reaction of a potential new drug with promising biological activity. The selected anticancer drug is thiogenistein—a new analogue of genistein. For the first time, a self-assembled monolayer of such a complex compound attached to the gold electrode is studied. Three instrumental techniques are applied in order to determine products of the TGE oxidation on the Au electrode: electrochemistry, IR-ATR, and MALDI-MS. Selected experimental results are interpreted with the use of molecular modeling and quantum mechanical density functional calculations. We hope that understanding simulated electrochemical reactions will help to determine key molecular fragments involved in the subsequent biochemical processes in living organisms. Apart from these physico-chemical studies, a preliminary evaluation of the anticancer activity of TGE is undertaken based on one human prostate cancer (DU145 line) and safety for normal prostate epithelial cells (PNT2 line).

The topicality of our research is emphasized by the works of Kim and Rizzo [21,22], who confirmed the importance of genistein as an anticancer drug via oxidative processes.

## 2. Materials and Methods

### 2.1. Materials

The reagents used in the study were obtained from POCh (Gliwice, Poland) and Sigma-Aldrich (Saint Louis, MO, USA). They were of the highest purity and used without prior purification. All solutions were prepared with deoxidized water, distilled, and cleaned in a “Milli-Q” filter apparatus (Millipore Corporation, Bedford, MA, USA). Its final resistance was 18.2 MΩ/cm.

TGE was manufactured in Łukasiewicz Research Network—Industrial Chemistry Institute (Łukasiewicz-ICHP), Warsaw, Poland. PBS buffer (the phosphate-buffered saline) was obtained from Syngen. The phosphate buffer was prepared from 0.2 M monobasic and dibasic sodium phosphate solution. The pH of the solutions used in the experiments was determined using a commercially available Mettler Toledo (Greifensee, Switzerland) pH meter. The solutions were deoxygenated with argon for 20 min before each experiment, while during the measurements gas was passed over the solution (argon with 99.5% purity from Air Products Kielce, Poland).

The monolayers of the TGE compound on the gold electrodes were prepared in the self-assembly process. First, the monolayers of the TGE compound were prepared on a gold surface by immersing the purified gold electrodes in ethanolic solutions containing 1 mM TGE. After their removal, the electrodes were rinsed thoroughly with ethanol, water, and ethanol to wash off the physically adsorbed molecules and left to dry in the air.

### 2.2. Electrochemical Measurements

Electrochemical experiments were performed using Autolab potentiostat (Eco Chemie BV, Utrecht, The Netherlands). The potentiostat was controlled using the GPES software. Electrochemical measurements were carried out in a three-electrode system. A silver/silver chloride (Ag/AgCl) electrode with saturated KCl was used as the reference electrode, the platinum plate was used as the auxiliary electrode. The working electrode was a gold electrode (Arrande, Werther, Germany) with a surface area of 0.61 cm^2^. The gold electrodes were 1.1 × 1.1 cm^2^ borosilicate glass plates on which gold was sputtered with a thickness of 200–300 nm. There was a chromium adhesive layer 2–5 nm thick between the gold layer and the glass. The electrolyte solutions were deoxygenated by bubbling argon (99.5% purity, Air Products, Kielce, Poland) through the solutions for 20 min. During the measurements, gas was passed over the solution. All experiments were performed at the temperature of 22 ± 2 °C.

### 2.3. IR Measurements

The infrared spectra were recorded on the Nicolet iS10 FT-IR spectrometer (Thermo Scientific, Waltham, MA, USA) using an ATR sampling module, on diamond crystals, in the range from 4000 to 650 cm^−1^, with the spectral resolution of 4 cm^−1^. For one spectrum 1000 scans were recorded. IR transmission spectrum of TGE was recorded in the KBr pellet in order to obtain the spectrum of the anisotropic sample.

### 2.4. Raman Spectroscopy Measurement

The FT Raman spectrum of TGE was recorded on the Nicolet NXR 9650 instrument (Thermo Scientific, Waltham, MA, USA) using a 1064 nm excitation from the Nd:YVO4 laser in the range from 3700 to 150 cm^−1^ with a spectral resolution of 4 cm^−1^. For one spectrum, 300 scans were recorded with 0.5 W of laser power.

### 2.5. MS Spectrometry

Mass spectra were acquired in positive reflector mode on Applied Biosystems/MDS SCIEX MALDI 4800 Plus TOF/TOF spectrometer (Matrix-assisted laser desorption/ionization time-of-flight mass spectrometry). 2,5-Dihydroxybenzoic acid (DHB), dissolved in CH_3_CN/H_2_O/TFA (50:50:0.1 ratio) was used as a matrix. Analyte ionization was achieved with 355 nm Nd:YAG laser firing at a 200 Hz rate. Laser fluence was within the 5500–6000 AU range. Typically, 1024 laser shots were accumulated. The acceleration voltage was set to 20 kV, lens to 10 kV, and the delayed extraction time to 250 ns. Raw spectra were analyzed and edited using the Data Explorer software, Version 4.9, Applied Biosystems.

### 2.6. Quantum Mechanical Modeling

Quantum mechanical modeling was performed using the density functional B3LYP method with medium-size Gaussian basis sets, i.e., 6-31G(d), 6-31G(d,p), and 6-311++G(d,p) for the H, C, N, O, and S atoms depending on the system investigated. Molecular geometry was optimized with the Berny algorithm implemented in the Gaussian G16 program [23]. The minimum was confirmed with all positive harmonic frequencies. For larger molecular systems the UFF molecular mechanics and the semiempirical quantum mechanical PM7 methods were used (also implemented in the G16 program). All calculations were performed on the HPC cluster in the Interdisciplinary Centre for Mathematical and Computational Modelling at the University of Warsaw, Poland.

### 2.7. In Vitro Study

DU145 (androgen-independent human prostate cancer) cells were grown in DMEM (Dulbecco’s modified Eagle’s medium, Sigma, Saint Louis, MO, USA), with the addition of 10% (*v*/*v*) FBS (fetal bovine serum, Sigma, Saint Louis, MO, USA). PNT2 (normal prostate epithelium) cells were maintained in RPMI 1640 medium (Sigma, Saint Louis, MO, USA) with 2mM glutamine and 10% FBS. Cells were cultured in a humidified atmosphere at 37 °C with 5% CO_2_, incubated until an 80% confluent cell monolayer was developed, and detached from the culture flasks using TrypLE™ Express (ThermoFisher, Waltham, MA, USA). Next, the obtained cell suspension was transferred into a 96-well experimental plate (Nunc, Roskilde, Denmark) at a density of 5 × 10^3^ cells/well. After 24 h, the cancer prostate epithelium was exposed to GE and TGE solutions (maximum content of DMSO was < 1%) which were added to the wells in five final concentrations: 200, 100, 50, 25, 12.5, and 6.25 μM. Cells were incubated for 6, 24, and 72 h in standard conditions, and after that, the viability, cytotoxicity, and morphology were determined. Cell viability was examined by resazurin-based reagent PrestoBlue^TM^ (ThermoFisher, Waltham, MA, USA). The assay was used to determine the mitochondrial metabolic activity by the reduction reaction of non-fluorescent resazurin to fluorescent resorufin. To determine the proliferation rate of DU145 cells and the cytotoxic effect of genistein and thioderivative, the ToxiLight^TM^ BioAssay Kit and the ToxiLight^TM^ 100% Lysis Reagent Set (Lonza, Basel, Switzerland) were used. The kit was used to quantify adenylate kinase (AK) in both the supernatant (representing damaged cells) and lysate (representing intact adherent cells). As a control, cells incubated with the growth medium containing 0.1% DMSO were used. The cell viability and cytotoxicity were evaluated following the manufacturer’s protocols for fluorescence or luminescence measurements using a microplate reader POLARstar Omega (BMG Labtech, Ortenberg, Germany). The results were expressed as the mean ± standard deviation (SD) from six samples for each experimental group.

Cell morphology and rate of proliferation were determined by staining the cells with hematoxylin and eosin solutions (Sigma-Aldrich, St. Louis, MO, USA) and observed under the fluorescence microscope (Olympus CX41, Tokyo, Japan).

## 3. Results and Discussion

### 3.1. Electrochemistry

In our previous article [5], we described experiments performed by cyclic voltammetry with free genistein dissolved in PBS buffer pH 7.4 using a glassy carbon electrode (GCE). In the first anode half-cycle, we observed the first peak at approx. 0.4 V which corresponded to the irreversible oxidation of the hydroxyl group on the C4′ carbon ring of genistein, while a second peak at approx. 0.8 V corresponded to the oxidation of hydroxyl groups on the C5, C7 carbon ring of genistein. The oxidation peaks decreased in the subsequent CV cycles, which was probably related to the adsorption of genistein oxidation products on the electrode surface.

In this article, the thiolated derivative of genistein, TGE was attached to a gold electrode. The presence of a thiol group allows for the spontaneous organization of TGE molecules in the course of the self-assembly process on the gold surface. As a result, a monolayer of this compound spontaneously forms on the gold electrode. Such systems have plausible advantages because nanomolar amounts of TGE in the monolayer can be studied without further adsorption of the compound in subsequent voltammetric cycles, as it was used in the previous studies with genistein dissolved in solution.

**Figure 2:**
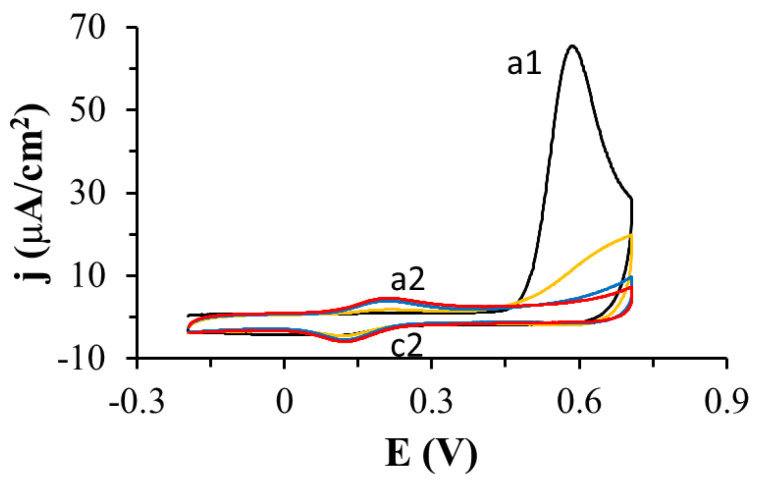
Cyclic voltammetric curves recorded with the gold electrode coated with thiogenistein in PBS pH 7.4, scan rate of the potential: 0.1 V/s, 1 (black curve), 2 (orange curve), 20 (blue curve), 30 (red curve) denote the cycle number.

**Figure 3:**
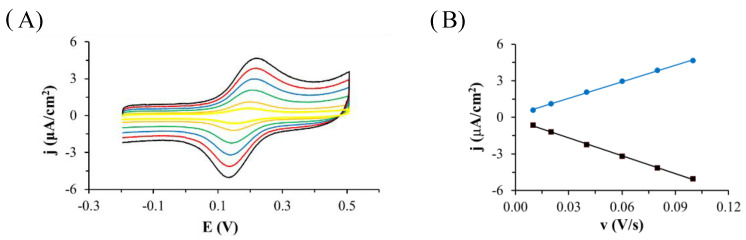
(**A**) Cyclic voltammetric curves recorded on the Au electrode modified with oxidized TGE monolayer (TGE_ox_) in deoxygenated PBS at pH: 7.4, scan rate of the potential 0.1 (black curve), 0.08 (red curve), 0.06 (blue curve), 0.04 (green curve), 0.02 (orange curve), 0.01 (yellow curve) V/s. (**B**) Dependence of the oxidation (blue dots and line) and reduction (black squares and line) peak current on the scan rate of potential.

The oxidation of TGE adsorbed on the gold electrode was investigated by cyclic voltammetry in PBS pH 7.4 (Figure 2). When the potential of the modified TGE electrode was varied from −0.3 V to 0.3 V, no Faraday current was observed in the voltammetric curves. If the potential was changed over a wider range of potentials, reaching more positive values, there was an irreversible oxidation peak at 0.59 V (peak a1) and a quasi-reversible pair of peaks at a potential close to 0.17 V (c2 and a2 peaks). After the first cycle, the a1 peak disappeared quickly and only a pair of c2 and a2 peaks was developed. The dependence of the c2 and a2 peak currents on the scan rate of potential was investigated (Figure 3). As the scan rate of potential increased, an increase in the peak current density of oxidation and reduction was observed on the voltammetric curves (Figure 3A). The current of the c2 and a2 peaks depends linearly on the scan rate of the potential in the range of 0.010–0.1 V/s, which indicates the presence of a system adsorbed on the gold electrode (Figure 3B). Similar voltammetric curves were recorded by other authors for the compounds 4-aminothiophenol and 4-hydroxythiophenol [24,25,26,27,28,29], as well as in the recent review from the Oliveira-Brett group [30]. The observed peaks were explained by the formation of dimers in the first stage, and then the formation of the quinone/hydroquinone system.

**Figure 4:**
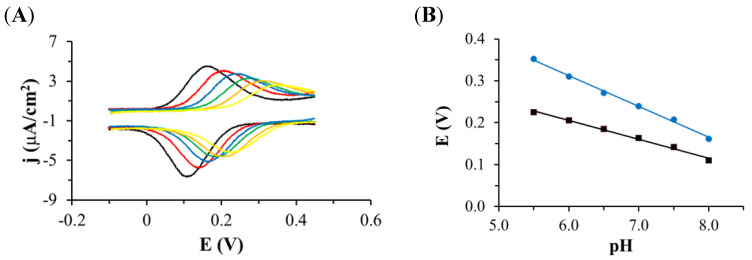
(**A**) Cyclic voltammetric curves recorded on the Au electrode modified with TGE in deoxygenated phosphate buffer pH: 8.0 (black curve), 7.5 (red curve), 7.0 (blue curve), 6.5 (green curve), 6.0 (orange curve), 5.5 (yellow curve), scan rate of the potential 0.1 V/s. (**B**) Dependence of the oxidation (blue dots and line) and reduction (black squares and line) peak potential on pH.

The properties of the electrode modified with oxidized TGE monolayer were also tested using cyclic voltammetry in solutions of different pH. The tests were carried out in phosphate buffer with different pH ranging from 5.5 to 8.0. The dependence of the a2/c2 peak potential on the solution pH was investigated (Figure 4). On the voltammetric curves, it can be observed that if the pH of the solution decreases, the pair of the a2/c2 peaks shifts to higher potentials, and the density of the peak current becomes smaller (Figure 4A). The relationship between the anode and cathode peak current density was determined and shown in Figure 4B. The slope of the curve for the cathode peak is about 45 mV per pH unit, which is close to 59 mV. The dependence of the potential of the a2 peak on pH is also linear, with the slope of the curve 74 mV per pH unit, which slightly deviates from the value of 59 mV. This slop indicated a two-electron and two proton redox process. This dependence may confirm the presence of a hydroquinone/quinone system in the B ring of genistein of hydroxyl groups C4′ and C3′ or C5′.

This is a very interesting observation because in this work the c2/a2 peaks appear only after the compound has been oxidized (a1 peak), which means that the hydroxyl group on the C4′ in the B ring of genistein does not oxidize in the first cycle. The a1 peak should be attributed to the oxidation of hydroxyl group on the C5 at the A ring of genistein because group C7 at the A ring of genistein is blocked by the linker with the thiol group. The a1 peak may also be responsible for the attachment of the -OH group in the B ring at C3′ or C5′ of genistein. In the B ring, a quinone/hydroquinone system is formed, which is manifested by the presence of a pair of a2/c2 peaks, which correspond to the 2e^−^/2H^+^ process. Additionally, the a1 peak is very large (peak current, charge under the peak) compared to the a2/c2 peaks which may suggest that more than one process is taking place there. We explain this by the simultaneous process of polymerization and the attachment of the -OH group to the B ring of thiogenistein.

**Figure 5:**
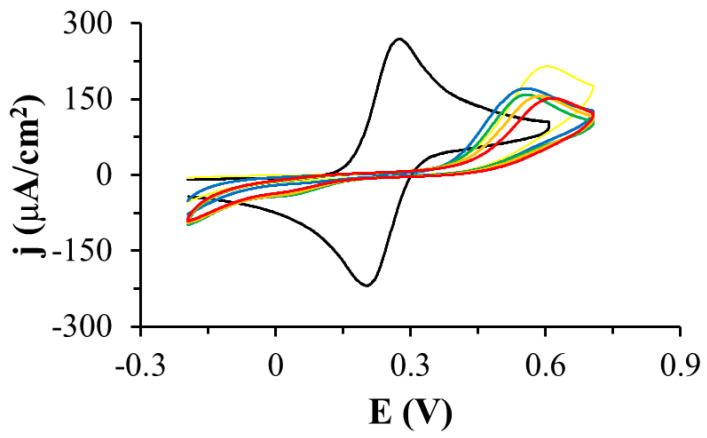
Cyclic voltammetric curves recorded using gold electrodes unmodified (black curve) and modified with TGE: 1 (yellow curve), 2 (blue curve), 10 (green curve), 20 (orange curve), 30 (red line), in deoxygenated 0.5 M KCl containing 1 mM Fe(CN)_6_^4−^, the scan rate of the potential 0.1 V/s.

In the next experiment, the quality of the TGE monolayer on gold was assessed in the presence of a fast [Fe(CN)_6_]^4−^ redox probe dissolved in the solution. The research was carried out in deoxygenated 0.5 M KCl containing 1 mM [Fe(CN)_6_]^4−^. Figure 5 shows cyclic voltammetric curves recorded with the use of the unmodified and modified TGE gold electrode in the potential range from −0.2 V to 0.6 V at the scan rate of the potential 0.1 V/s. During the recording of the first cycle, only the oxidation peak of the redox probe with a current density of 215 µA/cm^2^ and a potential of 0.60 V was observed on the voltammetric curve in the tested potential range. In this cycle, there is also oxidation of the monolayer (a1 peak described in the previous paragraph), which corresponds to the polymerization of TGE molecules and the attachment of the -OH group to the B ring. In the next cycle, the oxidation peak shifts to less positive potentials, and the peak current density decreased, which can be explained by the fact that the monolayer on the electrode is organized and the redox probe penetrates the monolayer less well. This may be due to the presence of a polymer on the electrode. In the third and subsequent cycles, the peak current density slightly decreases, and the peak potential shifts to more positive potentials, which proves that the monolayer is stabilizing.

**Figure 6:**
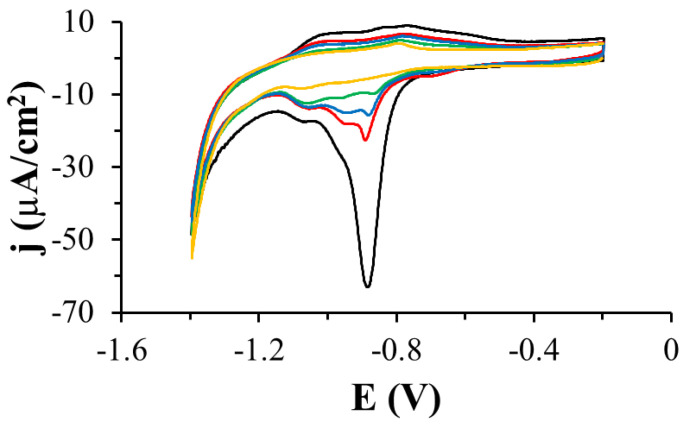
Cyclic voltammetric curves recorded for TGE-modified gold electrodes in a solution of 0.1 M NaOH, scan rate of the potential: 0.05 V/s, 1 (black curve), 2 (red curve), 3 (blue curve), 5 (green curve), 10 (orange curve) cycle.

The desorption of the TGE monolayer adsorbed on the gold electrodes was also performed during the registration of voltammetric curves in a wide range of potentials in 0.1 M sodium hydroxide solution (Figure 6). Reduction peaks in the potential range from −0.7 V to 1.2 V were observed on the voltammetric curves. Since there are more than one of these peaks, it suggests that the compound adsorbed on the electrode interacts with the electrode through the S-Au bond and that there are also quite strong intermolecular TGE-TGE interactions. This experiment confirms the presence of the TGE monolayer on the gold electrode.

In the case of studies of thiogenistein on the gold electrode, it is easier to interpret the results compared to the studies conducted with genistein dissolved in a solution, where there is strong adsorption of both the substrates and the products of electrode reactions. In the case of thiogenistein immobilized on the gold electrode, there is a monolayer of this compound on the electrode and redox processes are observed at the molecular level. Our observations and conclusions are consistent with those described by Oliveira-Brett and co-workers [30].

### 3.2. IR Spectroscopy

**Figure 7:**
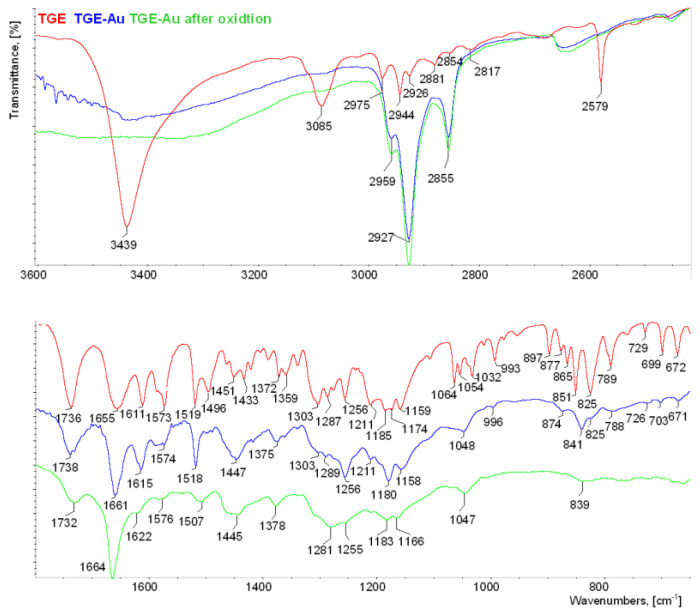
IR spectra of TGE (in KBr pellet), TGE on the Au electrode before and after oxidation (ATR). The upper window is in the range of 3600–400 cm^−1^; the lower window is in the range of 1800–650 cm^−1^.

The IR spectrum of the anisotropic sample of TGE is shown together with the Raman spectrum in Figure S1. For better clarity, three IR spectra of TGE (KBr) and IR-ATR spectra of TGE on the Au electrode before and after oxidation are collected in Figure 7.

The presence of hydroxyl groups in the TGE molecule is proven in the IR spectrum by the presence of the band at 3439 cm^−^^1^. The first distinctive difference between the TGE spectrum and both spectra of TGE on the Au electrode before after oxidation is in stretching vibrations of hydroxyl groups. In contrary to the TGE spectrum on other spectra, these vibrations give a broad absorption band from 3600 to 3200 cm^−^^1^. Taking into consideration that in the TGE molecule two hydroxyl groups participate in inter- and intra-molecular hydrogen bonding, the broadening of the band is observed in both spectra of TGE on the Au electrode before and after oxidation. This may suggest changes in the nature of these hydrogen bonds.

Bands from C-H stretching vibrations from aromatic rings and the methylene group of the TGE molecule are observed in the range from 3085 to 2817 cm^−^^1^. Exact wavenumbers of methylene stretching modes have been used to distinguish between all trans (crystalline-like) and a disordered (gauche rich, liquid-like) conformation of alkyl chains. For alkanethioles containing more than 10 carbons in chains, a generally observed trend is that wavenumbers of the methylene stretching asymmetric and symmetric vibrations increase from about 2918 to 2924 cm^−^^1^ and from about 2851 to 2855 cm^−^^1^ respectively when going from a crystalline-like to a disordered conformation of the alkyl chain. However, shorter chains also exhibit a spectral signature characteristic for a disordered conformation [11,12,13,14,15,16,17,18,19,20]. In spectra of TGE on Au electrodes before and after oxidation two characteristic, very intensive bands at 2927 and 2855 cm^−^^1^ resulting from methylene asymmetric and symmetric vibrations are observed, respectively. These bands are shifted 1 cm^−^^1^ towards higher wavenumbers in reference to their counterparts in the TGE spectrum. This shift does not prove crystalline-like order, like for model alkanethioles, because the TGE molecule is characterized by the short alkyl chain. In order to calculate the twist and tilt angles, the intensity values were used for bands originating from methylene asymmetric and symmetric vibrations as well as from C-O-C asymmetric stretching vibrations from spectra of TGE (in KBr pellet) and TGE on the Au (IR-ATR) [31]. The twist and tilt angles were 43° and 77°, respectively.

In IR and Raman spectra of TGE molecule, the band from stretching vibrations of S-H is observed at 2579 cm^−^^1^. The absence of this band in both spectra of TGE on the Au electrode before and after oxidation indicates the formation of a new bond directly via the sulfur atom on the surface of the Au electrode. Two carbonyl groups are present in the TGE molecule, one in the chain and the second in the ring. The comparison of two values 1736 and 1655 cm^−^^1^ with the reference value of 1647 cm^−^^1^ for the carbonyl group from GE molecule [5,32] allows assigning the band at 1655 cm^−^^1^ to the ring and the band 1736 cm^−^^1^ to the chain. It is supposed that the carbonyl group from the ring participates in the intramolecular hydrogen bonding with a neighboring hydroxyl group [5]. The band from carbonyl stretching vibration from the chain in the spectrum of TGE on the Au electrode is slightly shifted towards higher wavenumbers from 1736 cm^−^^1^ to 1738 cm^−^^1^ which may indicate a breaking of the C = O...H-S bond. But in the spectrum of TGE on the Au electrode after oxidation, the carbonyl stretching band is shifted into 1732 cm^−^^1^. The band from carbonyl stretching vibration assign to the ring in the spectrum of TGE on the Au electrode is shifted towards higher wavenumbers from 1655 into 1661 cm^−^^1^ what indicates a weakening of the intramolecular C=O–O-H hydrogen bond that can be caused by an increase in intermolecular interactions with the neighboring molecules. However, an increase in the relative intensity of this band is observed. In the spectrum of TGE on the Au electrode after oxidation, the carbonyl stretching band is observed at 1664 cm^−^^1^. It is supposed that this significant shift towards higher wavenumbers is caused by the breaking of the intramolecular C=O–O-H bond during the oxidation reaction. V. Crupi et al. [32] described changes in the Raman and IR spectra of genistein caused by its inclusion into β-cyclodextrins cavity. The changes were analyzed in detail in relation to intra- and intermolecular hydrogen bonds in the wavenumber range 1500–1800 cm^−1^. As a main result, a large high wavenumber shift (∼17 cm^−1^) of the C=O stretching mode has been observed in passing from uncomplexed genistein to inclusion compounds. It has been ascribed to the breakdown of the intramolecular hydrogen bond of genistein during inclusion phenomena and the formation of intermolecular host–guest H-bonds. This is in agreement with the rearrangement of the H-bond environments revealed by the analysis of the FTIR-ATR spectra of the O–H stretching vibration that is downshifted in with respect to pure genistein.

In IR and Raman spectra of TGE bands from 1611 to 1496 cm^−^^1^ mainly originate from C=C vibrations. The band at 1611 cm^−^^1^ in the TGE spectrum is shifted towards higher wavenumbers to 1615 cm^−^^1^ and 1622 cm^−^^1^ in spectra of TGE on the Au electrode before and after oxidation, respectively. It is worth observing that a higher shift is for the last spectrum. Moreover, the relative intensity of the band at 1622 cm^−^^1^ decreased. Similar behavior is observed for bands: 1573 cm^−^^1^ (TGE), 1574 cm^−^^1^(TGE on the Au electrode) and 1576 cm^−^^1^ (after oxidation) as well as for doublet of bands at 1519 cm^−^^1^ and 1496 cm^−^^1^ (TGE). Observations of the whole range of C=C stretching vibrations may indicate profound changes in three rings of oxidized TGE on the Au surface.

In IR and Raman spectra of TGE in ranges of deformation vibrations of methylene and hydroxyl group two separate doublets are observed at 1451 cm^−^^1^ and 1433 cm^−^^1^ as well as at 1372 cm^−^^1^ and 1359 cm^−^^1^, respectively. In both spectra of TGE on the Au electrode before and after oxidation, broad bands from deformation vibrations of methylene groups at 1447 cm^−^^1^ and 1445 cm^−^^1^ are observed, respectively. Similarly, in these spectra, broad bands from hydroxyl groups at 1375 cm^−^^1^ and 1378 cm^−^^1^ are observed.

In IR and Raman spectra of TGE, the doublet at 1174 cm^−^^1^ and 1159 cm^−^^1^ originate from C-O-C asymmetric stretching and C-OH stretching vibrations. The symmetric stretching vibrations of the C-O-C group are observed in the Raman spectrum at 993 cm^−^^1^ and 877 cm^−^^1^. In the spectrum of TGE on the Au electrode, the doublet from C-O-C asymmetric vibrations is at 1180 cm^−^^1^ and 1158 cm^−^^1^ whereas in the spectrum of TGE after oxidation the doublet is significantly shifted about 10 cm^−^^1^ into 1183 cm^−^^1^ and 1166 cm^−^^1^.

In the TGE spectrum, bands from C-H deformation out-of-plane vibrations from aromatic rings are at 851 cm^−^^1^ and 825 cm^−^^1^. In the range of these bands in the spectra, TGE on the Au electrode before and after oxidation bands at about 841 cm^−^^1^ and 839 cm^−^^1^ are observed, respectively. Moreover, it is worth noting that the band at 839 cm^−^^1^ is very broad and has smaller intensity than the band at 841 cm^−^^1^ what may prove that the oxidation of TGE influences rings of the molecule.

### 3.3. MALDI-TOF MS

**Figure 8:**
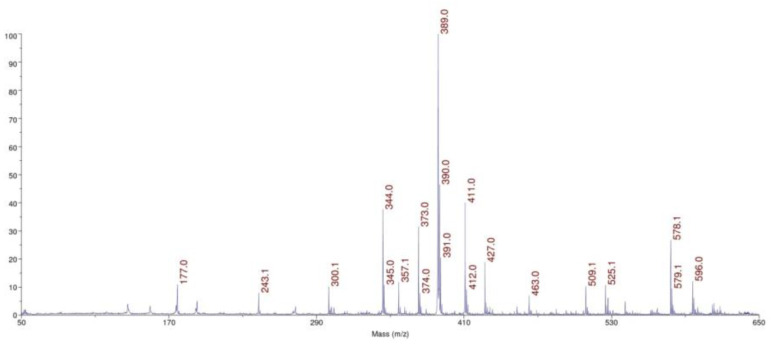
The MALDI-MS spectrum of the TGE compound on a steel plate in the DHB matrix.

**Figure 9:**
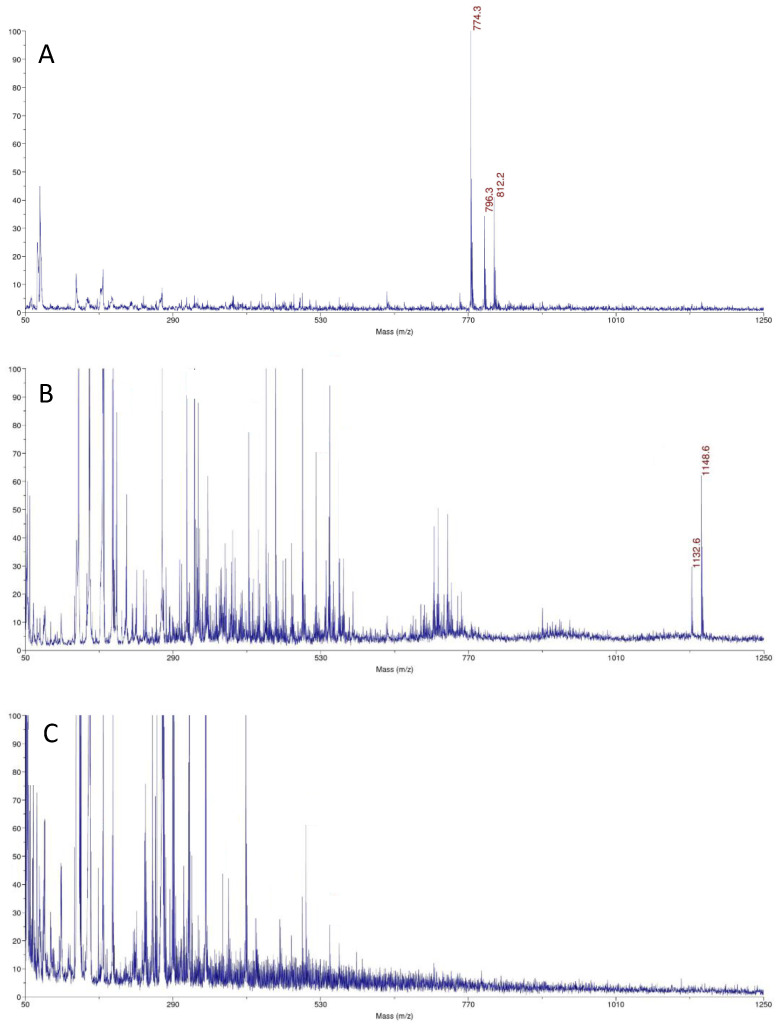
Comparison of MALDI-MS spectra: (**A**) TGE on the Au electrode before oxidation, (**B**) the product of oxidation on the Au electrode, (**C**) the Au electrode.

The small amounts of molecules are present on gold surfaces, so we use the ultrasensitive method to molecular surface characteristics. MALDI MS was used to characterize TGE SAMs on the gold electrode and the reactions on the monolayers. This technique was used to confirm the structure of the TGE compound, to identify TGE adsorbed on the gold electrode before oxidation and the product after oxidation. Taking into account the signal intensities and the quality of the spectrum, which are influenced by the type of matrix used, disturbing and interfering peaks from the blank (matrix), and the unmodified electrode, the DHB (2,5-dihydroxybenzoic acid) matrix has been turned out to be the best. In Figure 8, the spectrum of the TGE standard on a steel plate is presented. In Figure 9, the spectrum of the electrode with the compound before oxidation (Figure 9A), and the electrode with the compound after oxidation (Figure 9B), and the empty/clean electrode with the matrix (Figure 9C) are presented.

The TGE compound on the steel plate is observed at *m*/*z* 389 Da [M + H]^+^ ion in a positive ionization. Additionally, adducts with sodium and potassium are observed at *m*/*z* 411 Da [M + Na]^+^, *m*/*z* 427 [M + K]^+^, respectively. The *m*/*z* 774 Da ion is observed in the spectrum of TGE on the Au electrode before oxidation. The TGE is stamped from the monolayer on the electrode by the laser as a disulfide (Figure 9A), which agrees with literature data for thiol compounds [33]. The disulfide ions are not necessarily the products of photochemical reactions but may result from solvent extraction, for example during the application of matrix solution to the sample [34].

On the spectrum of TGE on the Au electrode after oxidation, two significant ions, *m*/*z* 1148 Da and 1132 Da, differing by 16 Da, are observed. The unexpectedly high molecular weight of the compound can indicate the presence, not only orobol, that was formed by adding an extra -OH group to the B-ring of genistein [35,36]. The mass of 1148 Da indicates the formation of a species which mass corresponds to the TGE trimer with additional modifications in the structure. The obtained results can indicate the formation of the trimer as a complex or condensation, which is known in the literature for tannins [37,38] or species that link the phenolic rings with the neighboring aryl ring via the C-O-C bond [39,40].

### 3.4. Molecular Modeling and the Quantum Mechanical Density Functional Calculations

**Figure 10:**
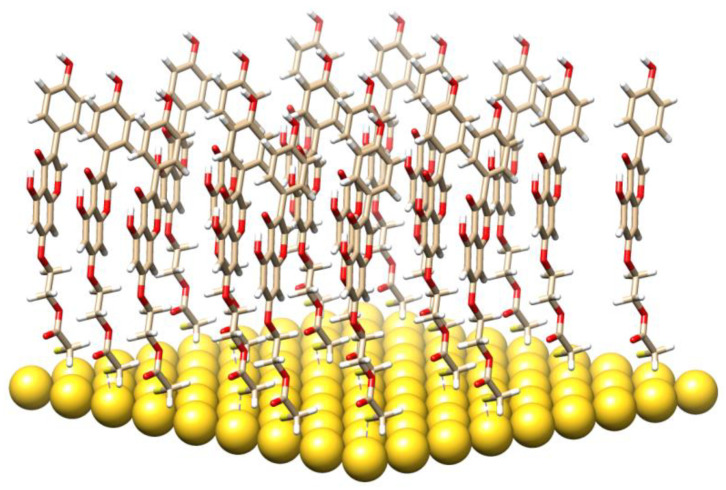
A molecular model of the TGE monolayer.

A molecular model of the TGE monolayer on the Au electrode plane is visualized in Figure 10. It is composed of 26 TGE molecules and 81 Au atoms. The 81 Au atoms are placed in the geometry corresponding to the fragment of the Au(111) plane. The TGE molecule was first optimized with the B3LYP/6-31G(d,p) method and then cloned over the Au plane and then reoptimized with the semiempirical PM7 method. The closest S-S distance was about 4.6 Å.

Selected properties of the TGE monolayer on the Au electrode were investigated with vibrational spectroscopy. The recorded IR and Raman spectra were described earlier in the text. The IR-ATR spectra were supplemented also with the theoretical quantum mechanical calculations of the harmonic frequencies and the intensities of the TGE molecule (Figure S2). The obtained geometry has an internal hydrogen bond between the C(5)-OH and O (at C-4). From a comparison of the IR spectra of the TGE and TGE on the Au electrode, one can deduce an increase of the wavenumber of the C(4)=O bond. To check the reason behind such a wavenumber increase the theoretical IR spectrum for a hypothetical structure without an intramolecular hydrogen bond (Figure S3). In such a structure, the rotated OH bond is characterized by the dihedral angle C(6)-C(5)-O-H equal to 0° in place of about 180° for the structure with the intramolecular H-bond. We found that the C(4)=O frequency increased after H-bond break by about 20 cm^−^^1^, i.e., more than twice as large than recorded in the IR spectra (only of about 6 cm^−^^1^), Table 1. This suggests that the intramolecular H-bond should be only slightly weakened and that this effect should originate from the intermolecular interactions with the neighboring molecules.

**Figure 11:**
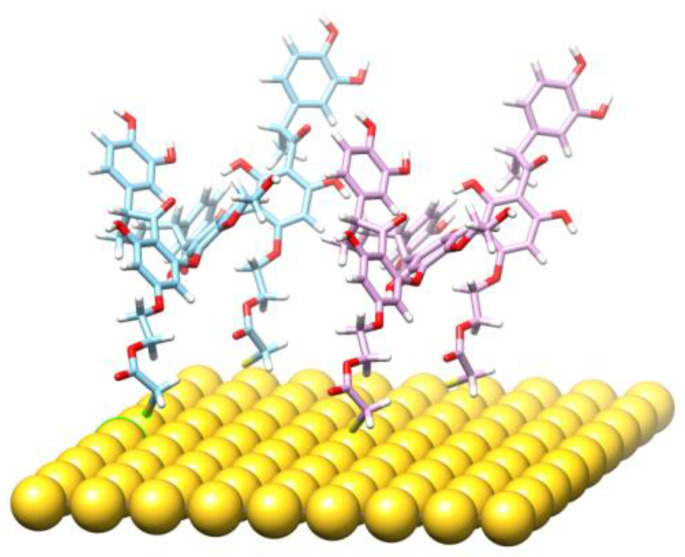
The theoretical model of a fragment of the TGE monolayer on the Au plane after oxidation.

A new product is created after TGE monolayer oxidation. Taking into account the results of electrochemical, spectroscopic and MS analyzes, we can propose a hypothetical model of a new product. There are some suggestions from the literature [41,42] that the electrochemical oxidation results in adding the -OH group to the B-ring of the genistein residue. The oxidation can also disintegrate the C-ring of genistein. Moreover, there are known species that link the phenolic rings with the neighboring aryl ring via the C-O-C bond [39,40]. Taking into account these facts, and the mass of the detected species, one can design a model of a hypothetical species possessing the following properties: high molecular weight, extra -OH group in the B-ring, disintegrated the C-ring, disintegrated thiolated linker of TGE, presence of the aryl-O-aryl linkage. The model is presented in the Figure 11 in order to make clear our point of view.

The sites suitable for an extra -OH group in the B-ring can be predicted based on the distribution of the spin density after the release of a proton from the 4′-OH group as well as from the 5-OH group, see Table 2.

Based on the spin density distribution, one can expect that the molecular positions at C3′ and C5′ in ring B (when a hydrogen atom is abstracted from the OH group at C4′) as well as at the C6 and C8 in ring A (when the hydrogen atom is abstracted from the OH group at C5) are particularly suited for new bond formation with neighboring molecules to form dimeric structure. Although the B3LYP and Hartree–Fock are essentially different theories, they both predict consistently the positions at the TGE rings which are suited for new bond formation.

In the oxidation process on the Au electrode, the TGE molecules in the monolayer can apart from C-ring disintegration also undergo a disintegration of the thiolated linker S-C(=O)O-CH_2_-CH_2_-(genistein core) from the middle molecule of the trimer which may result in releasing the thioglycolic acid residue. Here, we studied a model system to determine the energy output of such a process. The ΔG (the Gibbs Free Energy) of this model reaction is estimated to be about −5.3 kcal/mol following the B3LYP/6-311++G(d,p) calculations. Thus, one can expect that a split of the ester bond should be considered as one of the possible routes of TGE disintegration under system stress when forming a trimer on a monolayer.

Taking into account the molecular structure of TGE which involves the aromatic rings with the -OH groups as well as numerous cases in the literature [43] presenting the aryl ether linkages we proposed analogical linkages between the TGE molecules in the monolayer. To clarify our doubts on whether the C-C or C-O-C linkage can prevail a simple model was analyzed to compare directly the internal energy of isomers linked by the aryl-O-aryl and the aryl-aryl bond. Two isomeric structures (C_12_H_10_O_6_) formed by two 1,3,5-hydroxybenzene molecules were calculated with the B3LYP/6-311++G(d,p) method (Figures S4 and S5). Such a theoretical estimation corresponds to the isolated molecules, i.e., non-interacting with the environment. It appeared that the Gibbs free energy of both adducts is comparable.

### 3.5. In Vitro Study

**Figure 12:**
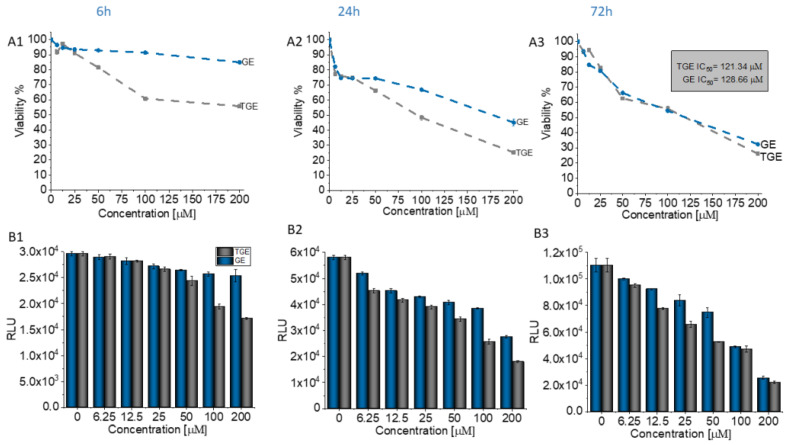

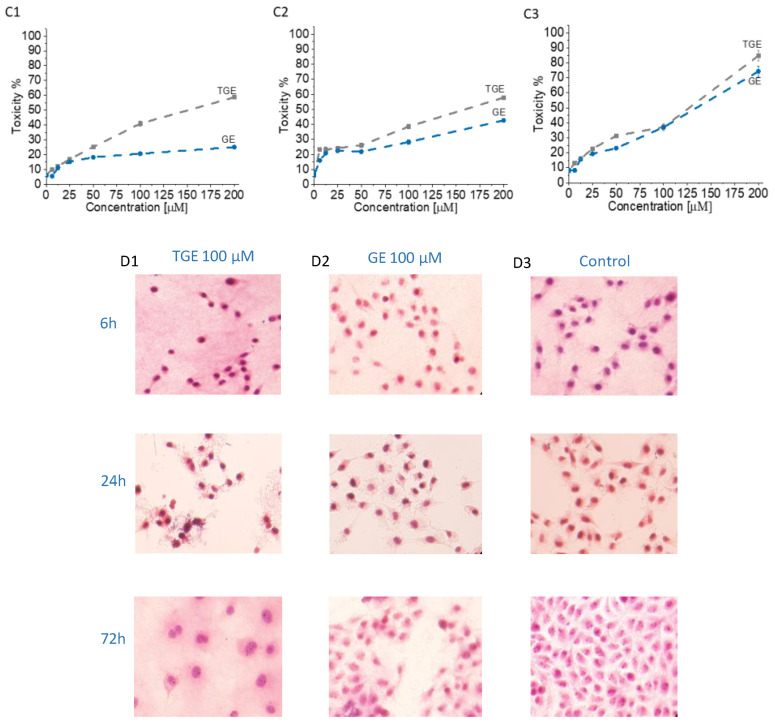
(**A1**–**A3**) Dependence of the viability (represented in % regarding control) on the concentration of TGE and GE incubated with DU145 cells for 6, 24, and 72 h. The viability was estimated based on the measurement of fluorescence intensity of the converted nonfluorescent resazurin to resorufin in metabolically active cells. (**B1**–**B3**) The luminescence intensity correlates with the adenylate kinase (AK) level obtained from all cells in the samples with ToxiLight 100% Lysate Control. The obtained results are proportional to the cell number of cells. (**C1**–**C3**) The cytotoxicity of the drugs was estimated based on the measurements of the AK level in the supernatant (released from the damaged cells) related to the AK level in the lysate (all cells in the samples). The data in (**A**–**C**) panels are representative of two independent experiments and are expressed as the mean ± SD. The error bars represent the ± SD. (**D1**–**D3**) Photomicrographs of the prostate cancer cells (DU145 line) morphology after 6, 24, and 72 h culture with the addition of 100 µM of TGE and GE as compared with the untreated group (control).

Genistein is one of the isoflavones whose anti-cancer potential is extensively studied. It modulates various steps of the cell cycle, apoptosis, angiogenesis, and metastasis in different types of cancers. Therefore, the number of studies on the synthesis and biological evaluation of new genistein derivatives is increasing every year [3]. In our research, we focused on a new thioderivative of genistein and its biological properties. A preliminary study of the anti-tumor activity of thiogenistein (TGE) was carried out on DU145 prostate cancer cells to determine the properties of the new derivative and compared to genistein as the base compound. The concentration-dependent cell viability and cytotoxicity effect induced by TGE and GE (as a reference) were measured after 6, 24, and 72-h of incubation (Figure 12). After the addition of 50 µM TGE solution, the viability of prostate cancer cells is reduced to 81.49% (±0.73%) and 66.12% (±1.30%) after 6 and 24 hours of incubation, respectively. However, in the case of GE, after 6 h, cell viability drops only to 92.91% (±1.01%), and after 24 h to 74.32% (±0.75%). The obtained results suggest that TGE reduces the viability of prostate cancer cells faster compared to GE. No meaningful differences are observed at lower concentrations. However, the most significant differences between cells were observed after 6 h (Figure 12A1). The addition of 100 μM of the TGE solution reduces the viability of cancer cells to 60.8 % (±0.55%), relative to the untreated cells. In contrast, for GE, cell viability remains high at 91.3% (±0.26%). The results achieved by the ToxiLight^TM^ assay are complementary to the analysis described above. The toxicity of TGE is shown in Figure 12B (compared with the effect of GE). The cytotoxicity for the three highest concentrations of TGE increased over time and reached 84% (±3.41%) after 72 h. In the case of the thiol derivative, a significant slow down in the proliferation of DU145 cells is observed (Figure 12B1–B3). The number of cells (estimated by ToxiLight 100 % Lysis Control) after 6, 24, and 72 h of incubation with the highest concentrations of TGE did not multiply. Based on these results, it could be concluded that TGE in a short time has far more negative effects on cellular respiration mechanisms and cytochromes of prostate cancer cells compared to GE. Additionally, the incubation of prostate cancer cells with TGE significantly affects their shape (Figure 12D1–D3). After only 6 h, cells become more rounded in contrast to GE-treated cells, which retain their characteristic, elongated shape. Due to early research, we are unable to determine the exact mechanism of TGE activity, which is responsible for reducing the rate of proliferation and reducing the viability of human prostate cancer cells. 

**Figure 13:**
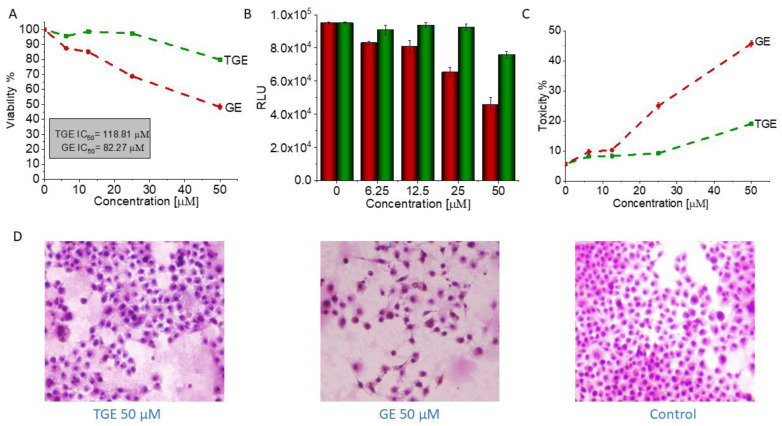
(**A**) The viability of PNT2 cells treated by different concentrations of GE and TGE for 72 h (determined by PrestoBlue^TM^ test). (**B**,**C**) The cytotoxicity effect of TGE and GE was evaluated based on the luminescence intensity of the supernatant and lysate. (**D**) The morphology of prostate epithelial incubated cells with 50 μM of GE and TGE for 72 h. The data in (**A**–**C**) panels are representative of two independent experiments and are expressed as the mean ± SD. The error bars represent the ± SD.

In addition to its anti-tumor properties, the new derivative should also show relative safety concerning healthy cells. Therefore, the initial characterization of TGE was supplemented with studies carried out on the PNT2 cell line—normal prostate epithelial cells. Figure 13 shows the concentration-dependent effect of TGE and GE on prostate epithelial cells. After 72 hours of incubation, the viability of PNT2 incubated with 50 µM GE decreased to 48.35% (±1.89%). With TGE, cell viability only drops to 79.88% (±0.86%). The results achieved by the ToxiLight^TM^ assay are complementary to the analysis described above. The cytotoxicity of TGE against normal prostate epithelial cells is 19.07% (±0.50%), while GE is as high as 45.75% (±1.09%) (Figure 13C). This is clearly visible in the photomicrographs (Figure 13D), which demonstrate the morphology and the number of prostate epithelial cells. After 72 h, cells incubated with 50 µM GE lose the normal epithelial morphology and an arrest of proliferation is evident, relative to the control group. In the case of cells incubated with TGE, the changes in these two parameters are not observed in comparison to untreated cells. Based on the obtained results, we can conclude that TGE shows both a lower cytotoxic effect against normal prostate epithelial cells and an increased antitumor activity. In the case of cancer cells treated with GE and TGE, significant differences in their viability are visible at the dose of 100 µM, while in the case of normal cells, these differences are already visible at the dose of 50 µM.

According to the literature, genistein is metabolized mainly through oxidation, sulfation, glucuronidation, hydroxylation, or methylation [44]. However, the influence of genistein metabolites on its anti-cancer properties is still not fully understood. Kiriakidis et al. in their work identified two genistein metabolites in T47D cells of the breast epithelium, such as 5,7,39,49-tetrahydroxyisoflavone (THIF)—orobol and 2 glutathinyl conjugates of THI [45]. THIF has also been shown to inhibit angiogenesis and the proliferation of endothelial cells. This suggests that THIF formation during genistein treatment may play a major role in cell cycle arrest, inhibition of cell proliferation, and activation of signaling pathways such as p38 MAPK that have been observed in T47D cells. According to the literature, over time, THIF oxidizes to o-quinone with the formation of hydrogen peroxides and reactive oxygen, which induces DNA strand breakage [46,47]. The synthetic derivatives, such as genistein glycosides, are also reported to possess anticancer activity when assessed in vitro. The anticancer potency of genistein glycosides varies depending on the sugar groups attached. For example, the addition of acetylated sugar hydroxyls to genistein resulted in more selectivity toward tumor cells. It is worthwhile to note that the anticancer potency of genistein and its derivatives differs in various types of cancer, depending on their selectivity toward the target molecule [48].

In vitro studies would suggest that changing the hydroxyl for an ether containing an -SH group on the C7 carbon in ring A may increase the cytotoxic properties of TGE. This effect can be attributed to the presence of a highly reactive -SH group [40]. Additionally, the presence of the -SH group in the new substituent may be responsible for the correct thiol-disulfide balance and the associated oxidation-reduction potential of cells. A similar mechanism is observed for glutathione which is a natural antioxidant [49].

## 4. Conclusions

This work contributes to the design of a simple and feasible strategy for thio-compounds in nano-drugs for use in oncology. In our previous publication, we described the synthesis of the thiolated genistein analog (TGE). In this work, while continuing the research on this new, promising compound, we examined its properties after oxidation as a process related to the reduction of cancer proliferation. The properties of TGE and its oxidation products were studied on a model system, i.e., the TGE monolayer on the Au electrode. In the course of the voltammetric experiments, it was proven that TGE is electrochemically active. The TGE monolayer undergoes irreversible oxidation reaction of the hydroxyl group in the A ring and the attachment of the -OH group to the B ring in the first stage, and then the quasi-reversible process of the quinone/hydroquinone system in the next stage. The formation of electroactive products that undergo redox reactions were observed. Electroactive centers of TGE were identified and its oxidation mechanisms were analyzed. The structure of TGE with a greater number of hydroxyl groups and additionally in a polymer configuration were discussed through MALDI-TOF MS and IR. Molecular modeling and the quantum mechanical density functional calculations supported this discussion. Our observations from the voltammetric experiments, supported with spectrometric data, are consistent with the literature regarding natural phenolic antioxidants described by the group of Oliveira-Brett [30]. In a preliminary in-vitro study, it was recognized that TGE has a higher cytotoxic activity towards DU145 prostate cancer cells and is safer for normal prostate epithelial cells (PNT2) than genistein (GE) itself. Moreover, the formation of TGE trimers upon oxidation deduced from the present study may enhance the biological response than the parent drug due to the synergizing of this response [50,51,52]. Most often, dimeric/trimeric drugs are not released (or cleaved) within the cell, so they can act as a completely new molecular unit inside the targeted cells [52]. The TGE monolayer firmly bound to the Au surface described in the present work may contribute to the design and development of the carriers of medicines in nanotechnology of biological applications.

## Figures and Tables

**Table 1:** Comparison of the IR C(4)=O modes in the TGE and Au-TGE systems, in cm^−^^1^.

System	IR	B3LYP	Comments
TGE	1655	1689	B3LYP for H-bonded TGE, Figure S2
Au-TGE	1661	1709	B3LYP for broken H-bond, Figure S3
Difference	6	20	-

**Table 2:** Theoretical prediction of the spin density distribution of the TGE molecule after hydrogen atom removal from the OH group in rings B or A. The calculations were performed with the B3LYP/6-311++G(d,p) density functional theory and the Hartree–Fock/6-311++G(d,p) theory (H-F).

Atom	Ring B		Atom	Ring A	
	**B3LYP**	**H-F**		**B3LYP**	**H-F**
O(at C4’)	0.609	0.899	O(at C5)	0.624	0.928
C4’	−0.089	−0.692	C5	−0.148	−0.673
C3’	0.231	0.784	C5-4 ^(^*^)^	0.16	0.792
C5’	0.221	0.781	C6	0.311	0.748
C2’	−0.134	−0.819	C7	−0.116	−0.689
C6’	−0.133	−0.833	C8-1	−0.113	−0.722
			C8	0.272	0.729

^(^*^)^ at the border of the fused A and C rings.

## Supplementary Materials

The following are available online at https://www.mdpi.com/article/10.3390/ijms22168783/s1.
