# Supplementary material for: Anti-Cancer and Electrochemical Properties of Thiogenistein—New Biologically Active Compound
Source: Int J Mol Sci. 2021 Aug 16;22(16):8783. doi: 10.3390/ijms22168783 (PMC8395759; doi:10.3390/ijms22168783)
Supplement: Supplementary file 1 [file ijms-22-08783-s001.zip › ijms-1307067-supplementary.pdf]

Supporting Information

Anti-Cancer and Electrochemical Properties of Thiogenistein—New Biologically Active Compound

Elżbieta U. Stolarczyk[1,*], Weronika Strzempek[2,3], Marta Łaszcz[1], Andrzej Leś[4], Elżbieta Menaszek[3], Katarzyna Sidoryk[5] and Krzysztof Stolarczyk[4]

[1] Analytical Department, Research Analytics Team, Łukasiewicz Research Network—Industrial Chemistry Institute, 8 Rydygiera Street, 01-793 Warsaw, Poland; marta.laszcz@ichp.pl

[2] Faculty of Chemistry, Jagiellonian University, 2 Gronostajowa Street, 30-387 Krakow, Poland; weronika.strzempek@doctoral.uj.edu.pl (W.S.)

[3] Faculty of Pharmacy, Collegium Medicum, Jagiellonian University, 9 Medyczna Street, 30-068 Krakow, Poland; elzbieta.menaszek@uj.edu.pl

[4] Faculty of Chemistry, University of Warsaw, 1 Pasteura Street, 02-093 Warsaw, Poland; ales@chem.uw.edu.pl (A.L.); kstolar@chem.uw.edu.pl (K.S.)

[5] Department of Pharmacy, Cosmetic Chemistry and Biotechnology, Chemistry Group, Łukasiewicz Research Network—Industrial Chemistry Institute, 8 Rydygiera Street, 01–793 Warsaw, Poland; katarzyna.sidoryk@ichp.pl

*Correspondence: elzbieta.stolarczyk@ichp.pl; Tel.: +48-22-568-25-81

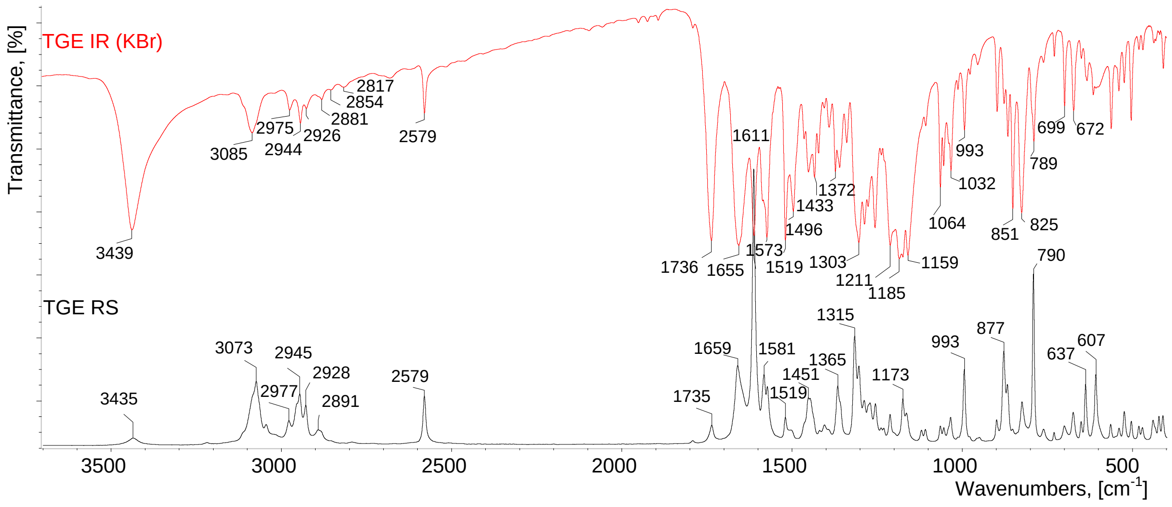

Fig. S1. IR (red) and Raman spectra (blue) of TGE.

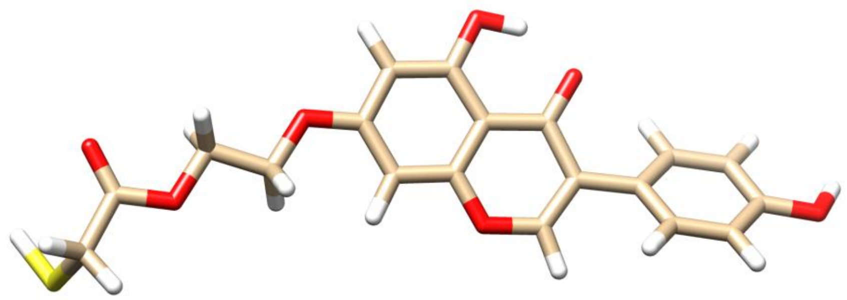

Fig. S2. The optimal geometry of the TGE molecule with the intramolecular hydrogen bond C(4)=O•••HO-C(5) following the B3LYP/6-311++G(d,p) calculations. The H(O at C-5)-O(at C-4) distance is 1.70 Å. Colors of atoms: H-white, C-gold, O-red, S-yellow.

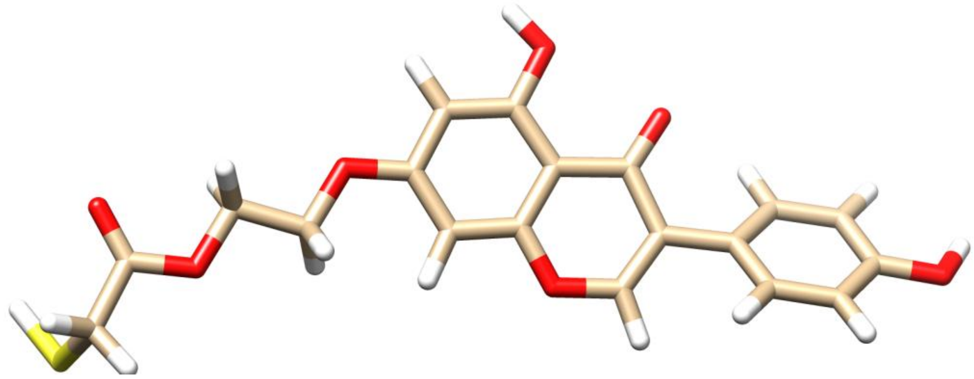

Fig. S3. The optimal geometry of the TGE molecule where the intramolecular hydrogen bond does not appear. The H(O at C-5)-O(at C-4) distance is 3.63 Å. Colors of atoms and the method of calculations as in the Figure S2.

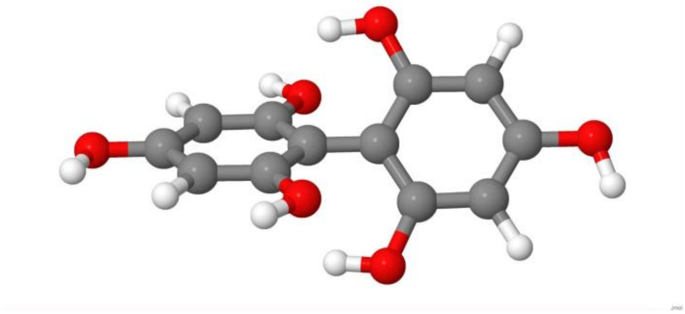

Fig. S4. The optimal geometry of the adduct of the 1,3,5-trihydroxybenzene linked by the C-C bond, following the B3LP/6-311++G(d,p) calculations.

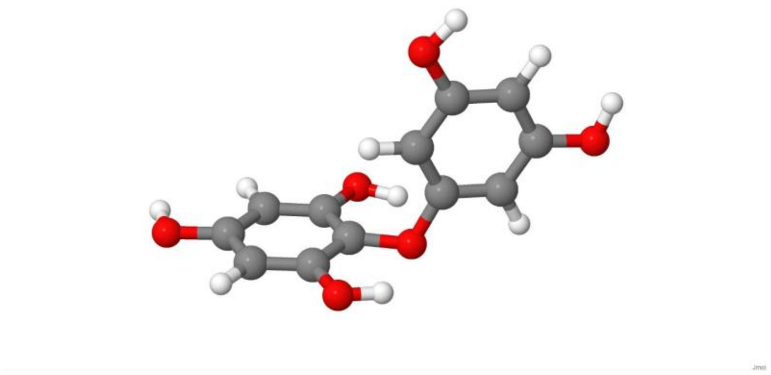

Fig. S5. The optimal geometry of the adduct of the 1,3,5-trihydroxybenzene linked by the C-O-C bond, following the B3LP/6-311++G(d,p) calculations.
